# Medico-Chirurgical Society of Edinburgh

**Published:** 1888-03

**Authors:** 


### MEDICO-CHIRURGICAL SOCIETY OF EDINBURGH.

Dr. JOHN PLAYFAIR showed the lungs of a child with a large pin impacted in the left bronchus. The little patient, of 15 months, was said to have swallowed a pin. Various unsuccessful attempts had been made at home and in the Royal Infirmary, to determine the whereabouts of the pin. Soon after admission into the Sick Children's Hospital, the child was seized with grave dyspnœa, necessitating tracheotomy. This relieved the patient much, and in due time it was dismissed apparently well. About a year later the resident physician was asked to go to see the child, which he found dead. The cause of death was cancrum oris; but on *post-mortem* examination a large pin was found impacted in the left bronchus, not far from the bifurcation of the trachea.

Mr. SYMINGTON showed a beautiful section to illustrate the anatomy of the ear and naso-pharyngeal connections. Among other points of interest it demonstrated that the antrum does not communicate directly with the internal meatus, but with that passage through the medium of the infundibulum. The section also gave further support to the view that the Eustachian tube is normally a closed tube.

*The Place of Specialism in General Practice, with Reference to Diseases of the Eye, Ear, Throat, and Nasal Cavities.*—Dr. GEORGE HUNTER (Linlithgow) read an elaborate paper on the above subject. By means of illustrative cases, he showed the value which attached to a fair working knowledge of these diseases, more especially in the case of the general practitioner, and he advanced a strong plea for their fuller study in our medical schools.

